# Impact of cable lock distribution on firearm securement after emergent mental health evaluation: a randomized controlled trial

**DOI:** 10.1186/s40621-024-00541-1

**Published:** 2024-11-11

**Authors:** Bijan Ketabchi, Michael A. Gittelman, Yin Zhang, Wendy J. Pomerantz

**Affiliations:** 1 Division of Emergency Medicine, Dept of Pediatrics, Children’s Hospital of Philadelphia, Perelman School of Medicine at theUniversity of Pennsylvania, 3501 Civic Center Blvd Colket Translational Research Building, 3rd Floor, Philadelphia, PA USA; 2 Division of Emergency Medicine, Dept of Pediatrics, Cincinnati Children’s Hospital Medical Center, University of Cincinnati College of Medicine, Cincinnati, OH USA; 3 Division of Emergency Medicine, Dept of Pediatrics, Cincinnati Children’s Hospital, University of Cincinnati College of Medicine, Cincinnati, OH USA; 4 Division of Biostatistics and Epidemiology, Cincinnati Children’s Hospital, Cincinnati, OH USA

**Keywords:** Firearm, Lethal means, Suicide prevention, Mental health, Emergency

## Abstract

**Background:**

Suicide-related presentations to pediatric emergency departments (PED) have increased in recent years. PED providers have the opportunity to reduce suicide risk by counseling on restricting access to lethal means. Supplementing lethal means counseling (LMC) with safety device distribution is effective in improving home safety practices. Data on PED-based LMC in high-risk patient populations is limited. The objective of this study was to determine if caregivers of children presenting to PED for mental health evaluation were more likely to secure all household firearms if given cable-style gun locks in addition to LMC.

**Methods:**

In this randomized controlled trial, caregivers completed a survey regarding storage practices of firearms and medication in the home. Participants were randomized to receive LMC (control) or LMC plus 2 cable-style gun locks (intervention). Follow-up survey was distributed 1 month after encounter. Primary outcome was proportion of households reporting all household firearms secured at follow-up. Secondary outcomes included: removal of lethal means from the home, purchase of additional safety devices, use of PED-provided locks (intervention only), and acceptability of PED-based LMC.

**Results:**

Two hundred participants were enrolled and randomized. Comparable portions of study groups completed follow-up surveys. Control and intervention arms had similar proportions of households reporting all firearms secured at baseline (89.9% vs. 82.2%, *p* = 0.209) and follow-up (97.1% vs. 98.5%, *p* = 0.96), respectively. Other safety behaviors such as removal of firearms (17.6% vs. 11.8%, *p* = 0.732), removal of medication (19.1% vs. 13.2%, *p* = 0.361), and purchase of additional safety devices (66.2% vs. 61.8%, *p* = 0.721) were also alike between the two groups. Both groups held favorable views of PED-based counseling. Within the intervention group, 70% reported use of provided locks. Preference for a different style of securement device was the most cited reason among those not using PED-provided locks.

**Conclusions:**

PED-based LMC is a favorably-viewed, effective tool for improving home safety practices in families of high-risk children. Provision of cable-style gun locks did not improve rate of firearm securement compared LMC alone—likely due to high baseline rates of firearm securement and preference for different style of lock among non-utilizers.

**Clinical Trial Registration:**

ID: NCT05568901. Clinicaltrials.gov. https://clinicaltrials.gov/. Retrospectively registered October 6, 2022. First participant enrollment: June 28, 2021.

## Introduction

Firearms are now the leading cause of death among youth in the United States (US)((National Center for Health Statistics (NCHS) 2022). In 2020 alone, 3,135 children and teens died from firearm-related injuries (National Center for Health Statistics (NCHS) 2022). Over the last decade, pediatric suicides have increased by 50%, with firearm-related suicides specifically, increasing nearly 75% (National Center for Health Statistics (NCHS) 2022). In part due to their 90% mortality rate (Miller et al. 2004), firearms make up 45% of all pediatric suicide deaths, making them the new leading means of suicide death in those under 18 years of age (National Center for Health Statistics (NCHS) 2022).

Securement of firearms and ammunition is associated with an almost 80% decrease in the odds of death from firearm-related suicide as well as 85% decrease for unintentional injury (Grossman 2005). The American Academy of Pediatrics (AAP) recommends that all firearms in the home should be kept locked and unloaded with ammunition secured separately (Beidas et al. 2020). Unfortunately, less than 40% of American firearm-owning families follow this guidance leaving approximately 4.6 million children living in households with loaded and unlocked firearms (Azrael et al. 2018). Given the impulsive nature of children and the accessibility of firearms, it is clear why 82% of firearms used in pediatric suicides belonged to a family member (Johnson et al. 2010). However, Monuteaux et al. estimated that if just 20% of households with even 1 unlocked firearm moved to locking all firearms within a year, up to 323 youth shootings, including 135 fatalities, could be prevented (Monuteaux et al. 2019).

Emergency providers are at the forefront of the pediatric suicide epidemic. Since 2007, pediatric emergency departments (PEDs) have seen a 2.5 fold increase in the number of suicide-related encounters (Kalb et al. 2019). PED providers have a unique opportunity to improve home safety for families of children at high risk for suicide or self-harm. Lethal Means Counseling (LMC) is a type of safety education that advises securement or removal of potentially hazardous household items such as medications, caustic cleaners, firearms, and other weapons. Several studies have demonstrated that counseling in health care settings can motivate families to improve home safety behaviors (Barkin et al. 2008; Albright and Burge 2003; Gittelman et al. 2006; Runyan et al. 2016; Uspal et al. 2021; Carbone et al. 2005). Moreover, when families receive tangible products, such as booster seats or home safety kits (Posner et al. 2004), they are more likely to make positive changes in home safety practices when compared to receiving education alone (Barkin et al. 2008; Gittelman et al. 2006; Uspal et al. 2021; Carbone et al. 2005). Similarly, firearm safety counseling without the provision of safety devices frequently results in subpar storage practices (Rowhani-Rahbar et al. 2016). While several studies have occurred in the primary care setting, there has been only one prior clinical trial (Uspal et al. 2021) directly comparing the impact of LMC with versus without the provision of firearm safety devices in a population that is high-risk for suicide or self-injury, such as those presenting to a PED for emergent mental health (MH) evaluation. However, examining the efficacy of LMC interventions in distinct geopolitical environments—Pacific Northwest (Uspal) vs. Midwest (our study)—could provide valuable insight for national initiatives. The objective of this study was to determine if provision of cable-style gun locks, in addition to LMC, improved self-reported securement of all firearms compared to LMC alone among caregivers of patients presenting to a PED for emergent MH evaluation.

## Materials & methods

### Study design and population

We conducted a single-center, prospective, randomized controlled trial within the PED of a free-standing tertiary care level-1 pediatric trauma center. Due to the nature of the study, investigators were not blinded to the interventions received by participants. This study was approved by the Cincinnati Children’s Hospital Medical Center (CCHMC) Institutional Review Board. Study protocol, counseling handout, and surveys are available upon request to study team.

The study population consisted of firearm-owning caregivers of children presenting to the PED for emergent MH evaluation. As only patients under the age of 18 can receive psychiatric care at our facility, all children of enrolled caregivers were less than 18 years of age. Caregivers were eligible for enrollment if they endorsed any firearms within the home and the patient resided in their home full- or part-time. Those who did not endorse firearms within the home, were unable to complete the English-based survey, and those within patient rooms deemed unsafe by study staff or behavioral safety team, were excluded. The patient’s disposition (admission vs. discharge) did not affect caregivers’ eligibility for enrollment.

### Survey development

Survey content was based on prior emergency department LMC for caregivers of pediatric patients (Runyan et al. 2016) and specific questions were created via expert opinion of PED physicians, injury prevention researchers, and psychiatric social workers. Pre-counseling surveys contained questions regarding caregiver demographics as well as firearm and medication storage practices. Follow-up surveys repeated questions about storage behaviors with additional questions regarding how participants viewed PED-based counseling, use of the provided locks, removal of lethal means from the home, and the purchase of additional safety devices after PED encounter.

Counseling handouts and surveys were piloted for readability and content among 12 firearm-owning caregivers (Sheatsley 1983). Inclusion criteria was revised to include all firearm-owning caregivers rather than only those with unsafely stored weapons. Potential participants preferred discussing storage practices of “all firearms” (versus answering questions around number of firearms and details about how each were stored) as well as relaying information anonymously via electronic survey as opposed to face-to-face encounter. Content regarding another lethal mean, medication, was added to reduce perceived judgement surrounding firearm ownership.

### Study procedure

All enrollment occurred within the primary PED of CCHMC between June 28, 2021 and February 10, 2022. Potential participants were identified by documented chief complaint of “Psychiatric Evaluation”, which includes, but is not limited to, those presenting for: suicidal ideation/attempt, homicidal ideation/attempt, aggression, behavior change, and hallucinations. The Psychiatric Intake and Response Center (PIRC) team is made up of social workers and attending psychiatrists that consult on all patients presenting to the PED for psychiatric evaluation. Caregivers who met inclusion criteria and received consultation by PIRC team were considered for enrollment. A convenience sample of caregivers were screened for enrollment by the study principal investigator (PI) or Clinical Research Coordinators (CRCs) specifically trained in study recruitment and lethal means counseling. CRCs were present in the PED 8am to 12am on weekdays and 11am to 9pm on weekends. The PI aided in recruitment, as needed, during times of high patient volumes.

Caregivers were approached for enrollment after patient had been evaluated by both emergency medicine and PIRC teams. If more than one caregiver was present, one caregiver was chosen by the family for participation. Participants provided electronic informed consent prior to completing survey questions. Neither consent nor assent was required from patients as no protected health information was collected. Eligibility screening and safety counseling with caregiver occurred outside of patient room, physically distant from patient, in order to avoid raising awareness of firearms in the home.

Enrollment occurred in parallel with a 1:1 allocation ratio. Enrolled caregivers were randomized into one of two study arms based on date of enrollment. Participants enrolled on odd-numbered dates were allocated to the control group (LMC alone), while participants enrolled on even-numbered dates were allocated to the intervention group (LMC + 2 cable-style gun locks). Participants completed the tablet-based survey with study team member present to clarify questions. The REDCap^®^ application was used for data collection and survey distribution.

The control arm of the study received standardized LMC from the study PI or CRC as well as a 1-page handout summarizing the counseling recommendations. The intervention arm received the same counseling and handout with the additional provision of 2 cable-style gun locks at no cost to caregivers. Counseling provided by study team was derived from the Suicide Prevention Resource Center’s “Counseling on Access to Lethal Means” training module (Suicide Prevention Resource Center 2019)  and the “Store It Safe” campaign (Store It Safe 2015) from the Ohio Chapter of the AAP. Guidance focused on securement of dangerous items in the household, such as medications and firearms, with locking devices—or more preferably removing these items from the home, even if temporarily. Particular attention was given to the AAP’s recommendations on safe storage of firearms, which state that all firearms in the home should be kept locked, unloaded, with ammunition secured separately (Store It Safe 2015). The cable locks were SnapSafe Cable Padlock (Item No. 75281). Cable locks were chosen as they have several advantages over other types of gun locks, including: (1) they are near-universally applicable to both handguns and long guns (2) cable locks are the least expensive type of gun locks, often costing 5-10x less than even the most basic safes/lockboxes (3) the possibility of unintentional discharge during securement process is much lower than that of devices such as a trigger locks and (4) even if a firearm is kept loaded, a cable lock will prevent the firing pin from striking a bullet/shell. Caregivers were provided with instructions included within the package of the provided gun locks and advised to store keys away from the firearm in a location inaccessible to their children. All patients and caregivers received usual care from PIRC team, including a standardized safety checklist and instructions on increased supervision of child. Counseling provided by study team members was supplementary to usual care.

A link to a follow-up survey was sent to participants 4 weeks after completion of baseline survey. This survey was distributed via text message using functionality within the REDCap^®^ application and sent to the mobile phone of the participant completing the initial survey. If a participant did not complete the follow-up survey, reminders were sent at 3-day intervals, up to 3 additional times. Participants received $10 gift cards upon completion of each survey.

### Outcome measures

The primary comparison groups were the “LMC Alone” control arm and the “LMC + Gun Locks” intervention arm. Pre-post analysis also occurred within each group. The primary outcome was self-reported securement of all firearms in the household with a locking device at 4 weeks post index PED encounter. Securement of all firearms with locking device was chosen as the primary safe storage outcome as it is the most protective factor against suicide and unintentional injury (Grossman 2005). For the purposes of this study, “secured” or “securement” is defined as firearms locked with/within a locking device (including but not limited to: cable lock, trigger lock, lockbox, gun safe, locked cabinet). Secondary outcomes included: caregiver acceptability ratings of PED-based counseling, change in proportion of households reporting all firearms & medications locked, removal of firearms & medications from the home, purchase of additional safety devices, and use of provided gun locks (intervention arm only).

### Statistical analysis

A sample size of 200 patients was determined based on a 95% confidence interval with an 80% power to detect a 30% difference in primary outcome—based on described outcome differences in previous clinic-based trials (Carbone et al. 2005). Sample size calculations accounted for an estimated 30% loss to follow-up, based on prior survey-based projects within CCHMC PED (Gittelman et al. 2006). Wilcoxon rank sum and Fisher’s exact tests were used to analyze differences between primary comparison groups at both baseline and follow-up, for continuous and categorical variables, respectively. Odds ratios and McNemar’s test were used to compare baseline vs. follow-up securement rates within and between study groups. Logistic regression—with adjustments for patient and caregiver age, gender and race—was used to compare change in proportions of caregivers reporting firearm securement between study arms.

## Results

**Fig. 1:**
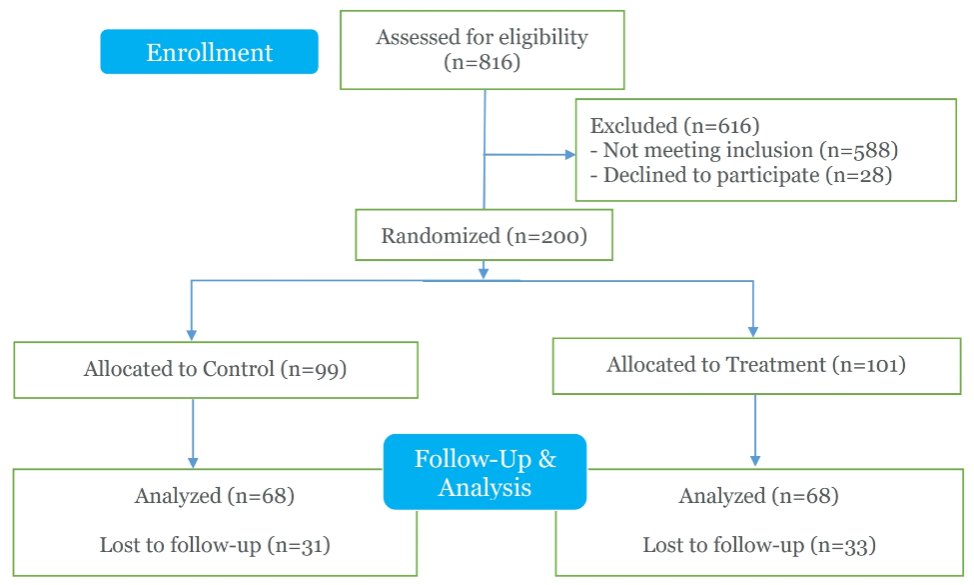
CONSORT flow diagram of study enrollment. Pre- vs. Post-Counseling firearm securement

During the study period, 3534 patients presented to CCHMC PED for emergent MH evaluations. A total of 816 caregivers were screened for eligibility, with 588 (72.1%) reporting firearms were not present in the home and 28 (3.4%) declining to participate. A total of 200 (24.5%) caregivers were enrolled and randomized into control and intervention groups (Fig. 1). Patients and caregiver demographics are shown in Table 1. Age and gender of patients are representative of overall population presenting to the CCHMC PED for MH evaluation; however there is a slight predominance of female patients in the intervention arm (49.5% vs. 59.4%).

**Table 1:** Patient and caregiver demographics

Patient characteristic	LMC alone (*n* = 99)	LMC + Gun Locks (*n* = 101)
Patient age in years [IQR]	14 [12, 15]	14 [11, 15]
Patient gender n (%)		
Female	49 (49.5%)	60 (59.4%)
Caregiver age n (%)		
30 or younger	5 (5.1%)	4 (4.0%)
31–40	41 (41.4%)	43 (42.6%)
41–50	38 (38.4%)	32 (31.7%)
51–60	11 (11.1%)	16 (15.8%)
61 or older	(4) 4.0%	6 (5.9%)
Caregiver gender n (%)		
Male	69 (69.7%)	80 (79.2%)
Caregiver race n (%)		
Black	10 (10.1%)	10 (9.9%)
White	83 (83.8%)	87 (86.1%)
Other/No Response	6 (6.1%)	4 (4.0%)

**Fig. 2:**
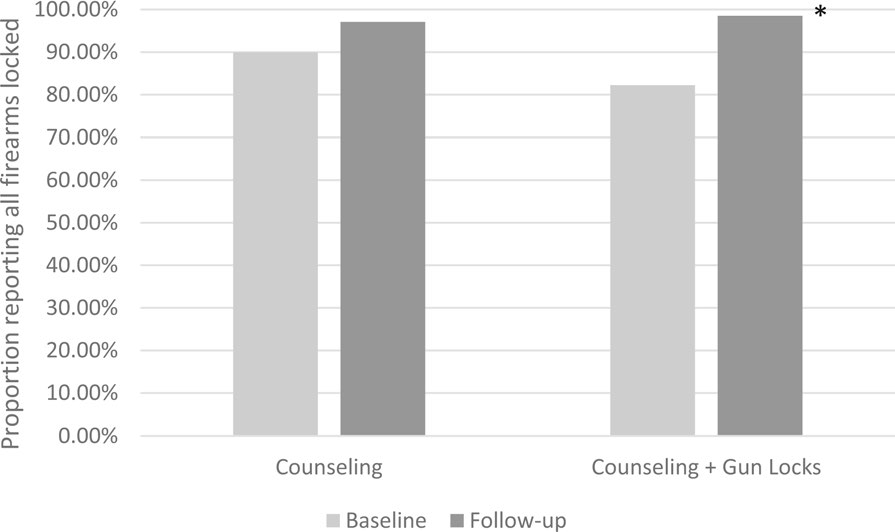
Change in proportion of caregivers reporting all firearms secured with locking device. *Indicates a significant difference from baseline. (Uspal et al. 2021)

Participants in both groups reported similar rates of securement of all firearms with locking devices at baseline (89.9% vs. 82.2%, *p* = 0.209). Comparable portions of participants within each study arm completed follow-up surveys; 68 (68.7%) in LMC Alone group and 68 (67.3%) in LMC + Gun Locks group. The post-encounter rates of firearm securement were nearly equivalent between the control (97.1%) and intervention (98.5%) arms (OR 2.03, 95% CI 0.19—44.27, *p* = 0.96) as shown in Table 2. The lock-receiving intervention arm had significantly higher odds of firearm securement at follow-up compared to pre-encounter baseline (OR 14.5, 95% CI: 2.9—264, *p* = 0.001); this was not true for the control arm (OR 3.7, 95% CI: 0.9—24.6, *p* = 0.103) (Fig. 2). The overall degree of change within each group (pre- vs. post-encounter) was not statistically different between the two study arms (*p* = 0.296).

**Table 2:** Securement of Lethal Means. Caregiver-reported securement of firearms and medications, pre-counseling (Baseline) and 4 weeks post-counseling (Four-week Follow-up)

Securement	LMC alone *n* (%)	LMC + Gun Locks *n* (%)	*P*-value	OR (95% CI)
Baseline n (%)	*n* = 99	*n* = 101		
All firearms secured	89 (89.9%)	83 (82.2%)	0.209	
All medication secured	44 (44.4%)	43 (42.6%)	0.957	
Four-week follow-up n (%)	*n* = 68	*n* = 68		
All firearms secured	66 (97.1%)	67 (98.5%)	0.960	2.03 (0.19–44.27)
All medication secured	59 (86.8%)	59 (86.8%)	1.000	1.00 (0.37–2.73)

Comparable outcomes were seen when examining medication storage practices. Rates of caregivers reporting medication securement at follow-up were identical between control (86.8%) and intervention (86.8%) arms (OR 1.0, 95% CI 0.37—2.73, *p* = 1.000). Additionally, both control (OR 8.8, 95% CI 3.1—20.9, *p* < 0.0001) and intervention (OR 8.2, 95% CI 3.8—19.4, *p* < 0.0001) arms had increased odds of medication securement, compared to their respective groups at baseline. Study groups displayed similarity in other safety behaviors at follow-up (Table 3). In the control group, 66.2% reported purchase of additional safety devices, compared to 61.8% in the intervention (*p* = 0.721). Comparable portions of each group removed firearms (17.6% vs. 11.8%, *p* = 0.732) and medications (19.1% vs. 13.2%, *p* = 0.361) from their homes.

**Table 3:** Other Safety Behaviors. Other caregiver-reported safety behaviors described on 4 week follow-up survey

Additional safety behaviors	LMC alone *n* (%)	LMC + Gun Locks *n* (%)	*P*-value
Firearms removed	12 (17.6%)	8 (11.8%)	0.732
Medications removed	13 (19.1%)	9 (13.2%)	0.361
Additional safety devices purchased	45 (66.2%)	42 (61.8%)	0.721

Within the intervention group, nearly 70% reported use of PED-provided locks to secure firearms in their home. Among the proportion not using the provided locks, the most common reasons were: (1) all firearms were already secured with locking device and (2) preferred a different style of safety device, such as safe or lockbox. The majority of participants from both groups had “very favorable” or “somewhat favorable” views of the counseling experience with 92.6% and 94.1% of the control and intervention arms (*p* = 0.738), respectively.

## Discussion

This randomized controlled trial of a high-risk pediatric patient population demonstrates the efficacy of LMC, but ultimately found that supplementing LMC with cable-style gun locks did not result in improved securement rates of all household firearms.

There are several possible reasons as to why our intervention did not impact storage practices. Both groups reported over 80% of households locking all firearms within the home. In the seminal case-control study by Grossman et al. examining storage practices among households of children who died by firearm-related suicide or unintentional injury, only 32% of case households reported storing firearms locked (Grossman 2005). There are a few possible reasons as to why our findings differed from that of Grossman. First, this low rate of securement only describes the storage practices of those who died, not those with suicidal ideation or other MH concerns. Second, nearly 80% of children who die by suicide were not receiving treatment for MH concerns (Ruch 2021)–differing from our population where many are well-connected to MH services and have received safety counseling on prior occasions. Our findings also corroborate a recent similar study examining firearm safety practices among caregivers of children with MH concerns at a PED or psychiatric hospital. These authors found that 74 − 85% of caregivers reported locking their firearms at all times (Uspal et al. 2021). Unlike our data, this prospective, pre-post study found that families who received no-or low-cost firearm safety devices had higher proportion of households reporting improved safety behaviors compared to those families that did not. The disparate findings are likely attributable to several reasons including but not limited to: different devices offered (cable locks vs. lockboxes), outcomes measured (securement of all firearms vs. triple safe storage), and/or the differing geopolitical environments (Pacific Northwest vs. Midwest) (Uspal et al. 2021).

One of the key findings of our study is that both groups’ rates of secure storage increased, each nearly reaching 100%. Another important outcome of our study is that over 92% of caregivers in both groups viewed the counseling in a positive manner. This is of particular significance given that fear of negative reaction by family is one of the top reasons pediatricians do not regularly counsel about firearm safety (Ketabchi et al. 2021). Our results should empower and encourage providers to discuss firearm safety, especially those treating patients at high risk for self-harm.

There are several limitations to our study. As with other prospective firearm studies, our results could be influenced by participation, recall, and/or social desirability biases. Those who agreed to participate may have been more likely to disclose firearm ownership, had better baseline safety behaviors, and/or a stronger predilection for behavior change than those who chose not to participate. Participants may be more likely to report positive safety behaviors due to recall bias or social desirability bias, especially during pre-counseling survey when study team was present. This is one possible explanation as to why a majority of the intervention arm reported use of PED-provided locks, despite nearly 80% previously reporting securement of all firearms at baseline.

While the within-group analysis found that only the intervention arm had an increased odds of households securing all firearms, the higher-than-expected baseline securement rates likely affected the ability to detect significant differences between groups. It is also possible that LMC alone may be enough to produce improved safety behaviors among this highly-motivated population, underscoring the importance of this timely counseling.

To best determine if access to affordable resources was the limiting factor in storage practices, we chose to focus our analysis on the issue addressed by cable locks: firearm securement. While this is the most protective storage factor, other components of safe storage, such as ammunition were not analyzed. Additionally, despite the benefits of cable locks described within the Methods section, firearm owners often prefer gun safes or lockboxes. This was also demonstrated within our study as preferring a different safety device was second most common reasons caregivers cited for not using the PED-provided cable locks. Unfortunately, the price of these items was cost-prohibitive for our study.

And lastly, as our study occurred at a single center, it is possible that perception of counseling and subsequent behavior change may differ in other regions of the country, limiting its generalizability.

## Conclusions

Our study demonstrates that the provision of cable gun locks did not result in a greater proportion of caregivers securing all firearms, compared to LMC alone. However, our findings illustrate the ability of PED-based LMC to produce numerous positive safety behaviors in households of children at elevated risk of self-harm. As firearms are now the leading cause of death in U.S. children—millions of whom live in homes with unsecured firearms—LMC should be considered standard of care for this high-risk patient population. Future studies will be needed to understand the needs of firearm-owning families and determine the efficacy of other devices and/or resources in producing improved home safety practices.

## Data Availability

Study data that support these findings are available on request from the corresponding author, BK. The data are not publicly available due to containing participant contact information that could compromise the privacy of research participants.

## Abbreviations

AAP: American academy of pediatrics
CCHMC: Cincinnati children’s hospital medical center
CRC: Clinical research coordinators
LMC: Lethal means counseling
MH: Mental health
OR: Odds ratio
PED: Pediatric emergency departments
PI: Principal investigator
PIRC: Psychiatric intake and response center
